# Emergency department crowding negatively influences outcomes for adults presenting with asthma: a population-based retrospective cohort study

**DOI:** 10.1186/s12873-022-00766-7

**Published:** 2022-12-24

**Authors:** Yifu Huang, Silvia S. Ortiz, Brian H. Rowe, Rhonda J. Rosychuk

**Affiliations:** 1 Department of Pediatrics University of Alberta, Edmonton Clinic Health Academy, Rm 3-524, 11405 87 Avenue NW, T6G 1C9 Edmonton, Alberta, Canada; 2 Department of Emergency Medicine, College of Health Sciences, University of Alberta, Edmonton, Alberta, Canada; 3 School of Public Health, College of Health Sciences, University of Alberta, Edmonton, Alberta, Canada

**Keywords:** Emergency department, Time to physician initial assessment, Length of stay, Crowding metrics, Asthma, Administrative data

## Abstract

**Background:**

Access to emergency department (ED) services is important for patients with acute asthma; however, ED crowding may impact the quality of care and compromise outcomes. We examine the association between ED crowding metrics and individual patient outcomes for adults presenting with asthma.

**Methods:**

This population-based retrospective cohort study extracted all ED presentations made by patients aged 18 to 55 years to 18 high-volume EDs in Alberta from April 2014 to March 2019. Physician initial assessment (PIA) time and ED length of stay (LOS) for discharged and admitted patients were calculated. Other metrics and patient outcomes were also obtained. Linear and generalized linear models were fit for continuous and categorical outcomes. Cox proportional hazards models were used for time-to-event outcomes.

**Results:**

There were 17,724 ED presentations by 12,569 adults. The median age was 33 years, and females (58.7%) made more presentations. ED crowding affected the PIA time for all triage groups. For the high acuity group (Canadian Triage and Acuity Scale [CTAS] 1/2), 1 h increase in median facility-specific PIA was associated with 26 min (95%CI: 24,28) increase; for the moderate acuity (CTAS 3) and low acuity (CTAS 4/5) groups, the individual-level PIA increased by 54 min (95%CI: 53,55) and 61 min (95%CI: 59,63), respectively adjusted by other predictors. Increases in facility PIA resulted in increase in odds of admissions for the high acuity group and increase odds of left without completion of care for the moderate and low acuity groups.

**Conclusion:**

The care provided for patients from all triage groups was impacted when EDs experienced crowding. Effective interventions are needed to mitigate ED crowding and improve care and outcomes for this important patient group.

**Supplementary Information:**

The online version contains supplementary material available at 10.1186/s12873-022-00766-7.

## Background

Emergency department (ED) crowding is a longstanding concern for the healthcare system in major economies [1–3]. Crowding is a state where the demand for emergency care services exceeds the capacity for providers to deliver timely and high-quality care [1–3]. Studies focussed on ED crowding have documented delays in time-sensitive interventions, poor patient outcomes, increased patient departures without completion of care, patient and provider dissatisfaction, increased health care costs, and even higher mortality [4–6]. These results were directly attributed to ED crowding, especially when hospital capacity was reached, and the physician initial assessment (PIA) was delayed [7]. While ED-based, these studies involved general ED presentations, and it is unclear how crowding affects care and outcomes for specific conditions.

Asthma is a common disease in North America. The prevalence of asthma among adults [8] in the U.S. is approximately 15%, and Canada has similar statistics [9]. Exacerbations of asthma can be life-threatening and exert additional pressure on hospital capacity in the absence of timely and evidence-based care [10]. Moreover, nearly 25% of the total asthma costs are attributed to acute asthma treatment in North America [11, 12]. Importantly, there are time-sensitive interventions that impact outcomes and delays in management during periods of ED crowding could have impacts on outcomes for these patients.

The objective of this study is to examine the association between crowding metrics in EDs and outcomes in adult patients presenting with acute asthma in the province of Alberta, Canada.

## Patients and methods

### Study design

This retrospective cohort study extracted data from population-based databases of patients residing in Alberta, Canada. No funding organization had any role in the conduct and reporting of this study.

### Study setting and population

All presentations acute asthma at the 18 highest-volume EDs during April 1, 2014, to March 31, 2019 were extracted for patients aged 18 to 55 years. The highest-volume EDs in Alberta were the focus for this project and were categorized into three groups: regional, urban, and academic/teaching.

### Study protocol

There were 6 administrative patient databases used for the study. The National Ambulatory Care Reporting System (NACRS) database collects information on ED presentations. Linkages to other large health administrative databases were made to provide other information for analysis. The Annual Cumulative Registry File (CRF) provided demographic and geographic information; the Discharge Abstract Database (DAD) provided inpatient hospitalization data; the Physician Claim File (PCF) provided physician follow-up visits; and the Alberta Vital Statistics (VS) provided death records. Community size and neighbourhood income level were provided by Statistics Canada based on postal code.

The NACRS database provided the dates and times of ED presentations, triage level, physician initial assessment (PIA) diagnostic and intervention data, and disposition status. We defined the start of the ED presentation as the minimum date-time between the patient registration and triage. Fiscal year, month and year, weekday/weekend, and shift time were defined by the ED presentation date-time. The International Classification of Diseases (ICD-10-CA) [13] was used to define the asthma cases and the study population. Since asthma diagnoses for ages greater than 55 may be inaccurate and ED research has found that patients above 55 years of age frequently have COPD [14], this project only includes patients diagnosed with asthma between 18 and 55 years of age. Triage was determined based on the Canadian Triage and Acuity Scale (CTAS) used in most Canadian EDs, which is used to indicate the level of acuity and urgency for each ED presentation [15, 16]. There are five levels of triage with associated PIA targets: CTAS 1 (Resuscitation): immediate (= 0 min); CTAS 2 (Emergent): ≤15 min; CTAS 3 (Urgent): ≤ 30 min; CTAS 4 (Semi-urgent/less urgent): ≤ 60 min; CTAS 4 (Non-urgent): ≤ 120 min. Ten disposition codes represent the patient’s status at the end of each ED presentation, and were grouped into six disposition categories: discharged (i.e., discharged home, discharged from program or clinic), admitted to hospital (i.e., admitted to critical care or operating room, admitted to regular ward), transferred (i.e., to another acute care facility, to another non-acute care facility), death (i.e., on arrival or in the ED), and left without completion of care (left without being seen [LWBS] or left against medical advice [LAMA]).

The CRF database provided demographic and geographic information on individual patients. Age was defined as the age at the date of the ED presentation and calculated based on the patients’ birthdate. Patient sex was coded as male or female. Using the postal code of the patients’ residence at fiscal year-end, patients were geo-coded into one of the province’s five health zones (North, Edmonton, Central, Calgary, South). Community size and neighbourhood income quintile were identified by linking postal codes and the 2006 census data, the Postal Code Conversion File Plus [17].

The PCF database provided data on follow-up visits, including the physician’s specialty, if any. Physician specialists of interest were coded as respiratory medicine specialists or other specialists. DAD database provided the start and end date-time of each patient’s hospitalization. The ICD diagnostic codes were used to determine the Charlson Comorbidity Score [18] and comorbidity indicator variables.

### Crowding metrics

Length of stay (LOS) and PIA time were calculated as ED crowding metrics [1] using data extracted from NACRS for patients presenting at any age for any condition (i.e., not restricted to adults or patients with asthma). For each ED presentation not ending in “left without completion of care”, the PIA time was defined as the difference between the presentation start time and the first physician assessment time. The first interaction for patients in Canadian EDs is with a nurse who performs triage. In the event that a patient is severe (CTAS 1 and some CTAS 2 cases), registration occurs after the PIA. Because, the PIA may occur before the reported ED start time in critical situations, negative values of PIA time were set as zero [19]. Each metric was calculated for each of the high volume ED facilities each hour within the same date during the study period. Hourly facility-specific ED crowding metrics (mean or median for skewed data) were calculated using all ED presentations within the same hour (e.g., 08:00–08:59) at the same facility. For each ED presentation, LOS was defined as the time from the start of ED presentation to the time of disposition; LOS for hospitalized patients and discharged patients were also calculated. Because the crowding metrics are continuous measures, they provide a measure of crowding on a continuum rather than classifying EDs into crowded or not crowded categories.

### Key outcome measures

Outcome variables were derived or calculated based on the original variables of the data sources and were broadly classified as duration time variables and categorical outcome variables. Duration time variables measure delays that potentially affect patient outcomes and categorical outcome variables measure patient severity and status at the end of ED presentation. Some outcomes were only applicable to subsets of the ED presentation data (e.g., outcomes measure for admitted or discharged patients only). Emergency department LOS was defined as the difference between the presentation start time and end time. For admitted patients, the end time was defined as the time of departure from the ED to an in-patient unit. For discharged patients, the end time was defined as the time of disposition decision [19]. The length of hospital admission was calculated as the time from the start of the hospitalization to the time of discharge.

For discharged patients, the time to next follow-up visit with a physician was defined as the number of days between the ED presentation and the next physician visit. The time-to-event variable was censored if: (1) the patient died within 183 days (i.e., 6 months) after the ED presentation and there was no follow-up physician visit after the visit (data censored on the date of death); (2) follow-up physician visit did not occur within 183 days. The time to next follow-up visit with a respiratory medicine specialist, and time to next follow-up visit with other specialists, were defined similarly for discharged patients. The time to next ED presentation was censored at March 31, 2019 or death date during the study period. Corresponding censoring indicators were developed for all time-to-event outcomes.

#### Data analysis

Numerical summaries (e.g., means and standard deviations [SDs]; medians and interquartile ranges [IQR]) and frequency distributions (percentages) described ED presentations. For medians, IQRs represent the 25th percentile and 75th percentile. Three CTAS groups were collapsed from the triage levels: high acuity for CTAS 1/ 2, moderate acuity for CTAS 3, and low acuity for CTAS 4/5. Linear and Cox proportional hazards (PH) models were applied for duration time variables, including time-to-event variables with censoring indicators, and generalized linear models used for categorical outcome variables. Models were applied to assess the association between the ED crowding metric and the outcome variables by CTAS groups. Random effects accommodated the multiple correlated data from the same patient and variation in each ED facility.

Each crowding metric variable was included in both the model for unadjusted estimates and the full models for adjusted estimates to assess its effects on outcomes. Other predictors included in the full models were: age, sex, zone, weekday/weekend, month of year, shift, fiscal year, ED category, income level (lowest and second lowest neighbourhood income quintile vs. others), community size (population ≥ 100,000 vs. others), and selected comorbidities (i.e., mild/moderate/severe liver disease, diabetes mellitus, cancer or metastatic solid tumor, myocardial infarction, congestive heart failure, renal disease, peripheral vascular disease, stroke).

In addition, estimates, odds ratios (ORs), hazard ratios (HRs), and associated 95% confidence intervals (CIs) were provided. A p-value (p) less than 0.05 was considered to be statistically significant. Statistical analyses were conducted in R [20] and SAS [21].

## Results

### Demographics

**Fig. 1:**
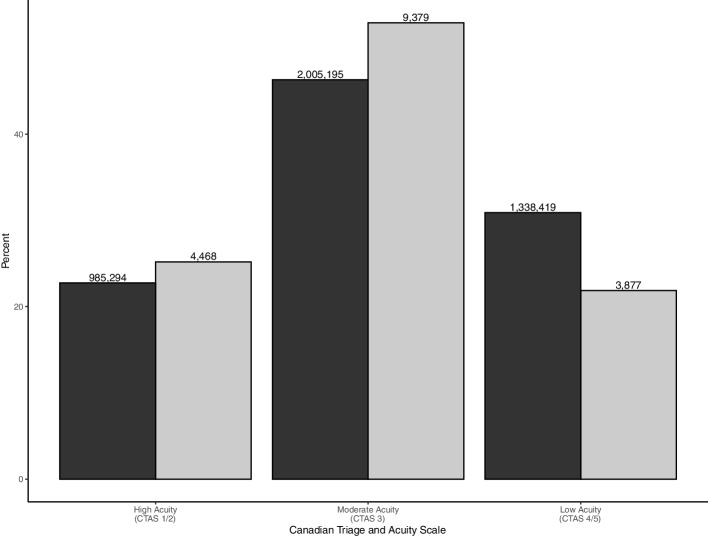
Percent of adult presentations with asthma and any condition by Canadian Triage and Acuity Scale (CTAS) groups. Dark gray indicates adult presentations with any condition and light gray indicates asthma presentations and. The numbers on top of the bars represent the count of presentations with asthma and all conditions by CTAS groups

There were 4,264,025 ED presentations made by adults for all conditions during the study period (Fig. 1); 17,724 (0.4%) presentations from 12,569 unique adult patients were made during the study period for acute asthma. There were 7 presentations deleted due to missing triage levels. The final dataset had 17,724 ED presentations (12,569 unique patients) for statistical analysis.

The median age was 33 years (IQR: 24.8, 42.5; Table 1), with more presentations made by females (58.7%). Approximately 60.6% of the presentations were from communities greater than 100,000 population, and 51.8% of presentations were made in the two main urban regions.

**Table 1:** Demographic and Emergency Department presentation characteristics for adults with asthma and by Canadian Triage and Acuity Scale groups

Characteristic	All Presentations (*n* = 17,724)	High Acuity (CTAS 1/2) (*n* = 4,468)	Moderate Acuity (CTAS 3) (*n* = 9,379)	Low Acuity (CTAS 4/5) (*n* = 3,877)
Sex, n (%)				
Female	10,400 (58.7)	2,629 (58.8)	5,628 (60.0)	2,143 (55.3)
Male	7,324 (41.3)	1,839 (41.2)	3,751 (40.0)	1,734 (44.7)
Age (years), median [IQR]	32.7 [24.8, 42.5]	34.5 [25.7, 44.4]	32.4 [24.7, 42.3]	31.7 [24.5, 40.9]
Zone of residence, n (%)				
North	1,592 (9.0)	169 (3.8)	846 (9.0)	577 (14.9)
Edmonton	5,305 (29.9)	1,883 (42.1)	2,459 (26.2)	963 (24.8)
Central	994 (5.6)	225 (5.0)	542 (5.8)	227 (5.9)
Calgary	7,053 (39.8)	1,759 (39.4)	4,337 (46.2)	957 (24.7)
South	2,747 (15.5)	423 (9.5)	1,178 (12.6)	1,146 (29.6)
Missing	33 (0.2)	9 (0.2)	17 (0.2)	7 (0.2)
Neighborhood income quintile, n (%)				
1 Lowest	3,788 (21.4)	1,005 (22.5)	2,009 (21.4)	774 (20.0)
2	3,430 (19.4)	853 (19.1)	1,804 (19.2)	773 (19.9)
3	3,015 (17.0)	714 (16.0)	1,603 (17.1)	698 (18.0)
4	3,068 (17.3)	662 (14.8)	1,714 (18.3)	692 (17.8)
5 Highest	2,261 (12.8)	509 (11.4)	1,203 (12.8)	549 (14.2)
Missing	2,162 (12.2)	725 (16.2)	1,046 (11.2)	391 (10.1)
Community size, n (%)				
≥ 100,000	10,754 (60.6)	3,032 (67.9)	6,016 (64.1)	1,706 (44.0)
10,000 to 99,999	4,142 (23.4)	555 (12.4)	1,979 (21.1)	1,608 (41.5)
< 10,000	753 (4.2)	178 (4.0)	387 (4.1)	188 (4.8)
Missing	2,075 (11.7)	703 (15.7)	997 (10.6)	375 (9.7)
ED category, n (%)				
Regional	5,240 (29.6)	733 (16.4)	2,493 (26.6)	2,014 (51.9)
Urban	9,188 (51.8)	2,662 (59.6)	5,184 (55.3)	1,342 (34.6)
Academic/teaching	3,296 (18.6)	1,073 (24.0)	1,702 (18.1)	521 (13.4)
Fiscal year, n (%)				
2014/2015	4,042 (22.8)	894 (20.0)	2,244 (23.9)	904 (23.3)
2015/2016	3,775 (21.3)	927 (20.7)	2,046 (21.8)	802 (20.7)
2016/2017	3,341 (18.9)	885 (19.8)	1,752 (18.7)	704 (18.2)
2017/2018	3,278 (18.5)	886 (19.8)	1,644 (17.5)	748 (19.3)
2018/2019	3,288 (18.6)	876 (19.6)	1,693 (18.1)	719 (18.5)
Month of year, n (%)				
January	1,432 (8.1)	343 (7.7)	737 (7.9)	352 (9.1)
February	1,254 (7.1)	300 (6.7)	669 (7.1)	285 (7.4)
March	1,338 (7.5)	344 (7.7)	690 (7.4)	304 (7.8)
April	1,346 (7.6)	318 (7.1)	719 (7.7)	309 (8.0)
May	1,456 (8.2)	342 (7.7)	774 (8.3)	340 (8.8)
June	1,358 (7.7)	335 (7.5)	723 (7.7)	300 (7.7)
July	1,478 (8.3)	390 (8.7)	790 (8.4)	298 (7.7)
August	1,577 (8.9)	401 (9.0)	852 (9.1)	324 (8.4)
September	1,990 (11.2)	524 (11.7)	1,090 (11.6)	376 (9.7)
October	1,678 (9.5)	452 (10.1)	866 (9.2)	360 (9.3)
November	1,328 (7.5)	342 (7.7)	720 (7.7)	266 (6.9)
December	1,489 (8.4)	377 (8.4)	749 (8.0)	363 (9.4)
Day of week, n (%)				
Weekday (Mon-Fri)	12,490 (70.5)	3,146 (70.4)	6,643 (70.8)	2,701 (69.7)
Weekend (Sat, Sun)	5,234 (29.5)	1,322 (29.6)	2,736 (29.2)	1,176 (30.3)
Time of day, n (%)				
Day (08:01–16:00)	6,493 (36.6)	1,496 (33.5)	3,372 (36.0)	1,625 (41.9)
Evening (16:01–24:00)	6,866 (38.7)	1,850 (41.4)	3,612 (38.5)	1,404 (36.2)
Night (00:01–08:00)	4,351 (24.5)	1,119 (25.0)	2,389 (25.5)	843 (21.7)
Charlson Comorbidity Score, median [IQR]	1.0 [1.0, 1.0]	1.0 [1.0, 1.0]	1.0 [1.0, 1.0]	1.0 [0.0, 1.0]
Charlson Comorbidities, n (%)				
Diabetes mellitus	748 (4.2)	286 (6.4)	364 (3.9)	98 (2.5)
Cancer	343 (1.9)	88 (2.0)	185 (2.0)	70 (1.8)
Mild/moderate/severe liver disease	174 (1.0)	50 (1.1)	89 (0.9)	35 (0.9)
Rheumatic	150 (0.8)	44 (1.0)	79 (0.8)	27 (0.7)
Congestive heart failure	139 (0.8)	72 (1.6)	56 (0.6)	11 (0.3)
Stroke	126 (0.7)	41 (0.9)	60 (0.6)	25 (0.6)
Renal	103 (0.6)	44 (1.0)	51 (0.5)	8 (0.2)
Peptic ulcer disease	69 (0.4)	21 (0.5)	30 (0.3)	18 (0.5)
Paralysis	41 (0.2)	17 (0.4)	21 (0.2)	3 (0.1)
Acute myocardial infarction	40 (0.2)	9 (0.2)	25 (0.3)	6 (0.2)
Peripheral vascular disorders	37 (0.2)	19 (0.4)	16 (0.2)	2 (0.1)
Dementia	36 (0.2)	12 (0.3)	20 (0.2)	4 (0.1)
HIV/AIDS	30 (0.2)	13 (0.3)	12 (0.1)	5 (0.1)

*CTAS* Canadian Triage and Acuity Score, *ED* Emergency Department, *IQR*  Interquartile range, *n*  Count, *HIV*  Human immunodeficiency virus, *AIDS* Acquired immunodeficiency syndrome

### Main results

Patients presenting to the Edmonton zone had higher acuity than the other zones (Table 1). Overall, 52.9% (9,379) of the ED presentations had an urgent (CTAS 3) triage level. The resuscitation (CTAS 1) and emergency (CTAS 2) triage levels accounted for 1.6% (278) and 23.6% (4,190) presentations, respectively (Fig. 1). There were 3,480 (19.6%) semi-urgent presentations (CTAS 4) and 397 (2.2%) ED non-urgent (CTAS 5).

Among all presentations, the vast majority (15,755, 88.9%) were discharged as expected. There were only three deaths that occurred for the high acuity group during the study period. Unsurprisingly, presentations that had less acute triage levels were less likely to be admitted. In the moderate and low acuity groups, 4.5% and 1.0% of the presentations were admitted, respectively, whereas 20.4% of presentations in the high acuity group were admitted.

The facility-specific hourly ED crowding metrics were calculated for all presentations of any condition. The median facility-specific PIA for all ED presentations was 1 h (hour) and 22 min (IQR: 48 min, 2h8min, Table S1).For adults presenting with acute asthma, the median facility-specific PIA was 1 h 6 min (IQR: 32, 124, Table 2). The high acuity group had shorter PIA (median = 36 min, IQR 18 min, 78 min) than other triage groups.

**Table 2:** Summaries for disposition outcomes, time to physician initial assessment and length of stay in Emergency Departments for all adults presenting with asthma by Canadian Triage and Acuity Scale groups

Outcome	All (*n* = 17,724)	High Acuity (CTAS 1/2) (*n* = 4,468)	Moderate Acuity (CTAS 3) (*n* = 9,379)	Low Acuity (CTAS 4/5) (*n* = 3,877)
Disposition, n (%)				
Discharged	15,755 (88.9)	3,378 (75.6)	8,634 (92.1)	3,743 (96.5)
Admitted	1,376 (7.8)	912 (20.4)	425 (4.5)	39 (1.0)
Transferred	110 (0.6)	72 (1.6)	36 (0.4)	2 (0.1)
Left without completion of care	477 (2.7)	101 (2.3)	284 (3.0)	92 (2.4)
PIA				
Median [IQR]	1h6m [32 m,2h4m]	36 m [18 m,1h17m]	1h19m [40 m,2h17m]	1h16m [39 m,2h13m]
Missing	1,345	275	652	418
ED LOS				
Median [IQR]	3h26m[2h13m,5h16m]	4h17m[2h44m,7h25m]	3h26m[2h16m,5h2m]	2h39m[1h36m,4h1m]
Missing	4,271	928	2,221	1,122
ED LOS for Discharged				
Median [IQR]	3h7m[2h2m,4h35m]	3h24m[2h23m,5h2m]	3h14m[2h10m,4h42m]	2h31m[1h32m,3h53m]
Missing	3,008	662	1,601	745
ED LOS for Admitted				
Median [IQR]	12h27m[7h17m,21h42m]	13h2m[7h14m,21h36m]	11h42m[7h24m,21h55m]	10h27m[7h35m,21h39m]
Missing	0	0	0	0

*CTAS *Canadian Triage and Acuity Scale, *ED* Emergency Department, *IQR* Interquartile range, *h* Hours, *LOS*  Length of stay, *m* Minutes, *n* Count, *PIA* Physician initial assessment

Among all 15,755 presentations ending in discharge, 7.6% (1,198) returned to the ED within 30 days; 34% (5,363) had a physician follow-up visit within seven days; 7.7% (1,207) had a respiratory medicine specialist follow-up visit within 30 days, and 5.6% (885) had a follow-up visit with other specialists within 30 days (Table 3).

**Table 3:** Outcomes after Emergency Department presentations for asthma ending in discharge by Canadian Triage and Acuity Scale groups

Outcome	All Discharged (*n* = 15,755 [88.9%])	High Acuity (CTAS 1/2) and Discharged (*n* = 3,378 [75.6%])	Moderate Acuity (CTAS 3) and Discharged (*n* = 8,634 [92.1%])	Low Acuity (CTAS 4/5) and Discharged (*n* = 3,743 [96.5%])
ED return within 30d, n (%)	1,198 (7.6)	361 (10.7)	641 (7.4)	196 (5.2)
Physician follow-up, n (%)				
Within 7d	5,363 (34.0)	1,210 (35.8)	2,946 (34.1)	1,207 (32.2)
Within 14d	7,426 (47.1)	1,641 (48.6)	4,129 (47.8)	1,656 (44.2)
Within 30d	9.756 (61.9)	2,148 (63.6)	5,397 (62.5)	2,211 (59.1)
Respiratory medicine specialist follow-up within 30d, n (%)	1,207 (7.7)	356 (10.5)	657 (7.6)	194 (5.2)
Other specialist follow-up within 30d, n (%)	885 (5.6)	239 (7.1)	493 (5.7)	153 (4.1)

*CTAS* Canadian Triage and Acuity Scale, *d* Days, *ED*  Emergency Department, *n* Count

For presentations ending in hospital admission (*n* = 1,376, Table 4) the median length of hospital stay was 3.0 days (IQR 1.8, 5.1). The median time from disposition decision to admission was 4 h 11 min (IQR 1 h 35 min, 13 h 33 min) and was, expectedly, longer for the high acuity group.

**Table 4:** Outcomes after Emergency Department presentations for asthma ending in admission by Canadian Triage and Acuity Scale groups

Outcome	All Admitted (*n* = 1,376 [7.8%])	High Acuity (CTAS 1/2) and Admitted (*n* = 912 [20.4%])	Moderate Acuity (CTAS 3) and Admitted (*n* = 425 [4.5%])	Low Acuity (CTAS 4/5) and Admitted (*n* = 39 [1.0%])
Length from disposition decision to admission				
Median [IQR]	4h11m [1h35m13h33m]	4h38m [1h38m,14h17m]	3h38m [1h29m,12h51m]	3h8m [1h30m,12h9m]
Length of hospital admission (days)				
Median [IQR]	3.0 [1.8,5.1]	3.3 [1.8,5.3]	2.8 [1.7,4.8]	2.6 [1.5,3.3]
Missing	9	6	3	0

*CTAS* Canadian Triage and Acuity Scale, *ED* Emergency Department, *IQR* Interquartile range, *h* Hours, *m* Minutes, *n* Count

**Fig. 2:**
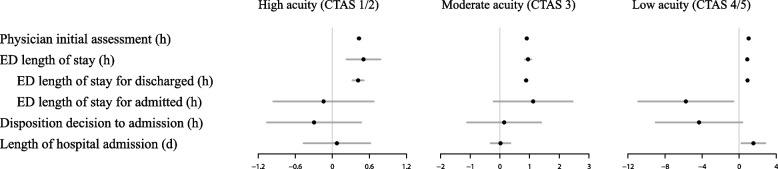
Adjusted coefficient estimates (with 95% confidence intervals) for ED crowding metric time to physician initial assessment (PIA) for each continuous outcome and each Canadian Triage and Acuity Scale (CTAS) group. Numerical values are provided in Table S2. CTAS Canadian Triage and Acuity Scale, d days, ED emergency department, PIA physician initial assessment

Individual-level PIA times for adults with asthma in all acuity groups increased as the median facility-specific PIA increased (Fig. 2, Table S2). For the high acuity group, a 1 h increase in median facility-specific PIA was associated with 26 min (95% CI: 24, 27) increase in individual-level PIA, adjusted by other predictors. For the moderate and low acuity groups, the individual-level PIA increased by 54 min (95% CI: 53, 55) and 61 min (95% CI: 59, 63), respectively, for every 1 h increase in median facility-specific PIA. Similarly, every 1 h increase in median facility-specific PIA was associated with 30 min (95% CI: 14, 47), 57 min (95% CI: 50, 63), and 52 min (95% CI 46, 58) increase in individual-level length of stay, for high, moderate and low acuity groups, respectively. For discharged patients, a 1 h increase in median facility-specific PIA, the individual LOS increased by 25, 53, and 54 min for the high, moderate, and low acuity groups, respectively. For the high acuity group, hospitalization was more likely with longer median facility-specific PIA (adjusted OR [aOR] = 1.13; 95% CI: 1.03, 1.23), adjusted by other predictors (Table S2). For the moderate acuity group, longer median facility-specific PIA was associated with a greater likelihood of leaving without completion of care (aOR = 1.48; 95% CI: 1.13, 1.95) and a lower likelihood of a return ED presentation within 30 days (aOR = 0.80, 95% CI: 0.67, 0.95).

When median hourly facility-specific LOS was used as a predictor, longer LOS was associated with a higher likelihood of admission in the high (aOR = 1.29; 95% CI: 1.23, 1.34) and moderate (aOR = 1.47; 95% CI: 1.31, 1.64) acuity groups (Table S3).

## Discussion

This large, population-based administrative data study extracted all 17,724 ED presentations for acute asthma made by patients aged between 18 and 55 years to 18 high-volume EDs during a five-year period. We described the characteristics of patients and ED presentations and examined the association between the PIA time, ED crowding time metric and patient outcomes. We found that ED crowding affects care differently based on patient acuity. For example, ED crowding appeared to generate less impact on high acuity patients with acute asthma. The PIA and overall length of stay increased the least for these patients; however, delays were associated with a higher likelihood of admission.

Conversely, patients with moderate acuity presentations were affected most by ED crowding as the increase in facility specific PIAs resulted in increased individual PIAs, LOS for all ED presentations, LOS for discharged patients, and a higher proportion of patients requiring admission. These results suggest that delays in timely, evidence-based care have a negative impact on outcomes for patients with acute asthma of moderate severity. Early management of patients with acute asthma using systemic corticosteroids [22], inhaled short-acting anti-cholinergic agents [23], intravenous magnesium [24], and inhaled corticosteroids [25] in the ED have been shown to reduce hospitalization in systematic reviews. Moderate acuity group patients also tended to have less relapse back to the ED. This finding is not surprising since moderate and low acuity patients have less severe disease and lower risk of relapse.

We also found that the patients’ odds of left without completion of care also increased proportionally to delay in facility-specific PIA for the moderate acuity group. Leaving without completion of care is considered an indicator of ED crowding and poor quality of care [26]. While some research has found that adult patients who leave the ED without completion of care tend to be at a lower risk of admission within seven days than patients assessed by a physician and later discharged [4], such data include all ED presentations, not a time-sensitive condition such as asthma. The time to PIA is also strongly correlated with the number of patients who leave the ED without being assessed by a physician [27]. Clearly, LWBS and LAMA outcomes are potentially dangerous, and reducing these events is important for conditions like asthma, where with the risk of poor outcomes is higher.

Finally, patients with low acuity presentations reacted differently to increasing evidence of ED crowding. In these cases, patients already had a low risk of being admitted; however, with increasing crowding, these patients reacted by more often leaving prior to the completion of care. Since the effectiveness of within-ED care is transient, out-patient treatment following discharge with systemic and inhaled corticosteroids is critically important to reduce relapse and improve outcomes. Patients who elect to LAMA may be at risk of not receiving the evidence-based care required for safe transition back into the community.

Asthma is a chronic respiratory condition and exacerbations of acute asthma are common, especially in patients with poorly controlled disease. Exacerbations can be triggered by non-adherence to evidence-based care, as well as exposure to airways irritants (e.g., perfumes, cigarette smoke, chemicals), the environment (e.g., air pollution, smoke from wildfires, etc.), and upper respiratory tract infections. These irritants result in airway inflammation and bronchospasm that results in symptoms of cough, wheezing and shortness of breath, which when severe or when alternative care is unavailable (e.g., holidays, weekends, and off hours) may necessitate ED presentation. Fortunately, treatments are effective especially in the ED setting. For example, systemic corticosteroids as well as short-acting beta-agonists and anticholinergic agents are effective treatments to relieve symptoms and prevent admission. These evidence-based and guideline–directed treatments, however, must be delivered in a timely manner to prevent adverse outcomes. As this study has demonstrated, when ED crowding delays physician assessment, the administration of these treatments is also delayed. When crowding is present and cannot be avoided, innovative strategies (e.g., nurse- or respiratory therapist-initiated protocols, early administration of systemic corticosteroids, asthma teams, etc.) to expedite care may be warranted.

Notwithstanding the large sample, population-based data and robust data linkages, this study has several limitations. First, these administrative datasets do not include data about the treatments, prescriptions, and recommendations that each patient received in the ED. The number of patients referred to a specialist is also not available. Second, the databases do not contain other information about smoking status, body mass index, diet, exercise, sleep and other factors known to impact asthma control. Further, although asthma is more common in Indigenous Peoples (*First Nations, Métis and Inuit) *[28] and may be an indicator of outcomes in some conditions, for a variety of reasons membership in any of the Indigenous communities in Canada was not requested. Further research in this area is warranted. Third, the results may not be generalizable to other areas with different healthcare systems because the study only focused on EDs in Alberta, a Canadian province where no barriers to access health care exist. Fourth, this study did not include patients who seek care from other health services. The study population may not represent all adults with asthma. Finally, there was no confirmation of the diagnosis of asthma; however, by mixing asthma with other diagnosis (e.g., COPD, bronchitis) this would reduce the chance of identifying differences. Overall, we do not believe any of these limitations invalidate the results presented here.

## Conclusions

Adult patients with acute asthma represent a small, albeit important, group of ED patients. While most patients experience excellent outcomes, some present with severe disease, and hospitalization occurs following 10% of presentations. Timely interventions exist to reduce hospitalization and improve outcomes; however, over the study period, these high volume EDs experienced periods of worsening crowding. When conditions deteriorate and delays increase, patients with acute asthma suffer unintended consequences. These EDs generally demonstrate flexibility to accommodate the most severe patients; however, during periods of crowding admissions increased, and low acuity patients often left prior to completion of care. Overall, general interventions designed to reduce ED crowding and efforts to deliver care in a timely manner to patients with acute asthma both appear warranted.

## Supplementary Information

**Additional file 1**: **Table S1**. Summaries of facility-specific hourlymetric median time to physician initial assessment and length of stay forpresentations for any condition and any age. **Table S2**. Regression estimates for linear models(beta coefs), odds ratios for logistic models (ORs), and hazard ratios for Coxproportional hazard models (HRs) with associated 95% confidence intervals (CIs)for Emergency Department (ED) crowding metric time to physician initialassessment (PIA) for each outcome and each Canadian Triage and Acuity Score(CTAS) group. **Table S3**. Regression estimates for linear models(beta coefs), odds ratios for logistic models (ORs), and hazard ratios for Coxproportional hazard models (HRs) with associated 95% confidence intervals (CIs)for Emergency Department (ED) crowding metric ED length of stay (LOS) for eachoutcome and each Canadian Triage and Acuity Score (CTAS) group.

## Data Availability

Data is the property of Alberta Health and the authors are not allowed to provide the data. Requests can be made for the same data from Alberta Health for researchers who meet the criteria for access to confidential data. Researchers are welcome to inquire for further information at Health.RESDATA@gov.ab.ca.

## Abbreviations

aOR: adjusted odds ratio
CI: confidence interval
COPD: chronic obstructive lung disease
CRF: Annual Cumulative Registry File
CTAS: Canadian Triage and Acuity Scale
d: day
DAD: Discharge Abstract Database
ED: emergency department
h: hour
HR: hazard ratio
ICD: The International Classification of Diseases
IQR: interquartile range
LAMA: left against medical advice
LOS: length of stay
LWBS: left without being seen
min: minute
n: count
NACRS: National Ambulatory Care Reporting System
OR: odds ratio
PCF: Physician Claim File
PIA: physician initial assessment
VS: Alberta Vital Statistics
