# Supplementary material for: Emergency department crowding negatively influences outcomes for adults presenting with asthma: a population-based retrospective cohort study
Source: BMC Emerg Med. 2022 Dec 24;22:209. doi: 10.1186/s12873-022-00766-7 (PMC9789669; doi:10.1186/s12873-022-00766-7)
Supplement: Supplementary file 1 — Additional file 1: Table S1. Summaries of facility-specific hourlymetric median time to physician initial assessment and length of stay forpresentations for any condition and any age. Table S2. Regression estimates for linear models(beta coefs), odds ratios for logistic models (ORs), and hazard ratios for Coxproportional hazard models (HRs) with associated 95% confidence intervals (CIs)for Emergency Department (ED) crowding metric time to physician initialassessment (PIA) for each outcome and each Canadian Triage and Acuity Score(CTAS) group. Table S3. Regression estimates for linear models(beta coefs), odds ratios for logistic models (ORs), and hazard ratios for Coxproportional hazard models (HRs) with associated 95% confidence intervals (CIs)for Emergency Department (ED) crowding metric ED length of stay (LOS) for eachoutcome and each Canadian Triage and Acuity Score (CTAS) group. [file 12873_2022_766_MOESM1_ESM.docx]

Table S1. Summaries of facility-specific hourly metric median time to physician initial assessment and length of stay for presentations for any condition and any age.

|  | All Years | 2014/2015 | 2015/2016 | 2016/2017 | 2017/2018 | 2018/2019 |
| --- | --- | --- | --- | --- | --- | --- |
| PIA, median [IQR] | 1h22m[48m,2h8m] | 1h24m[51m,2h11m] | 1h17m[46m,2h1m] | 1h17m[45m,2h1m] | 1h26m[50m,2h12m] | 1h25m[49m,2h14m] |
| LOS, median [IQR] | 3h32m[2h25m,5h1m] | 3h27m[2h22m,4h53m] | 3h27m[2h21m,4h53m] | 3h29m[2h23m,4h55m] | 3h39m[2h30m,5h9m] | 3h43m[2h31m,6h15m] |

m=minutes; h=hours; CTAS=Canadian Triage and Acuity Scale; ED=emergency department; IQR=interquartile range; PIA=time to physician initial assessment; LOS=length of stay.

Table S2. Regression estimates for linear models (beta coefs), odds ratios for logistic models (ORs), and hazard ratios for Cox proportional hazard models (HRs) with associated 95% confidence intervals (CIs) for Emergency Department (ED) crowding metric time to physician initial assessment (PIA) for each outcome and each Canadian Triage and Acuity Score (CTAS) group. Adjusted estimates are adjusted for age, sex, income quintile, community size, patient’s zone of residence, weekday, month of year, time of shift, fiscal year, ED category, and comorbidities (i.e., mild/moderate/severe liver disease, diabetes mellitus, cancer or metastatic solid tumor, myocardial infarction, congestive heart failure, renal disease, peripheral vascular disease, stroke).

| **Outcome** | | **High Acuity (CTAS 1/2)** | | **Moderate Acuity (CTAS 3)** | | **Low Acuity (CTAS 4/5)** | |
| --- | --- | --- | --- | --- | --- | --- | --- |
|  | | Unadjusted (95% CI) | Adjusted (95% CI) | Unadjusted (95% CI) | Adjusted (95% CI) | Unadjusted (95% CI) | Adjusted (95% CI) |
| PIA (hrs), beta coef^a^ | | 0.44 (0.42,0.47)* | 0.43 (0.40,0.46)* | 0.94 (0.92,0.96)* | 0.90 (0.88,0.92)* | 1.06 (1.04,1.09)* | 1.02 (0.99,1.05)* |
| ED LOS (hrs), beta coef | | 0.69 (0.42,096)* | 0.50 (0.23,0.78)* | 1.12 (1.01,1.23)* | 0.95 (0.84,1.06)* | 1.06 (0.96,1.15)* | 0.87 (0.77,0.97)* |
|  | Discharged^b^ | 0.53 (0.44,0.62)* | 0.42 (0.33,0.51)* | 1.03 (0.98,1.07)* | 0.88 (0.84,0.93)* | 1.09 (1.03,1.16)* | 0.90 (0.84,0.97)* |
|  | Admitted^c^ | -0.12 (-0.95,0.71) | -0.14 (-0.96,0.67) | 1.28 (0.00,2.55)* | 1.12 (-0.22,2.46) | -1.17 (-6.93,4.59)* | -5.75 (-10.89,-0.61)* |
| Disposition, OR | | | | | | | |
|  | Admitted | 1.10 (0.95,1.28) | 1.13 (1.03,1.23)* | 1.29 (1.05,1.60)* | 1.17 (0.92,1.50) | 0.86 (0.23,3.27) | 0.59 (0.05,6.65) |
|  | Left without completion of care | 1.01 (0.71,1.45) | 1.00 (0.68,1.47) | 1.51 (1.18,1.91)* | 1.48 (1.13,1.95)* | 1.53 (0.96,2.46) | 2.16 (1.02,4.59) * |
| Time to ED return (days), HR^b^ | | 0.94 (0.89,1.00)* | 0.97 (0.91,1.03) | 0.94 (0.90,0.98)* | 0.95 (0.91,1.00)* | 0.96 (0.90,1.03) | 0.98 (0.91,1.06) |
| ED return within 30 days, OR^b^ | | 0.93 (0.75,1.15) | 0.95 (0.76,1.19) | 0.80 (0.68,0.94)* | 0.80 (0.67,0.95)* | 0.79 (0.54,1.14) | 0.80 (0.53,1.21) |
| Time to first physician follow-up (days), HR^b^ | | 1.02 (0.99,1.06) | 1.00 (0.97,1.04) | 1.03 (1.01,1.05)* | 1.00 (0.98,1.03) | 1.06 (1.02,1.09)* | 1.01 (0.97,1.05) |
| Physician follow-up, OR^b^ | | | | | | | |
|  | Within 7 days |  | 1.03 (0.95,1.11) | 1.07 (1.02,1.12)* | 1.02 (0.97,1.07) | 1.15 (1.06,1.23)* | 1.05 (0.97,1.14) |
|  | Within 14 days | 1.10 (1.03,1.18)* | 1.04 (0.96,1.12) | 1.06 (1.02,1.11)* | 1.01 (0.96,1.06) | 1.12 (1.05,1.20)* | 1.02 (0.95,1.11) |
|  | Within 30 days | 1.06 (0.98,1.15) | 1.01 (0.93,1.09) | 1.07 (1.02,1.12)* | 1.02 (0.97,1.07) | 1.13 (1.05,1.21)* | 1.02 (0.94,1.10) |
| Time to first respiratory medicine specialist follow-up (days), HR^b^ | | 1.02 (0.96,1.10) | 0.97 (0.90,1.04) | 1.10 (1.05,1.16)* | 1.03 (0.98,1.09) | 1.15 (1.06,1.25)* | 1.00 (0.91,1.10) |
| Respiratory medicine specialist follow-up within 30 days, OR^b^ | | 1.13 (0.86,1.49) | 1.14 (0.84,1.55) | 0.94 (0.74,1.18) | 0.88 (0.69,1.14) | 1.12 (0.65,1.92) | 1.12 (0.59,2.14) |
| Time to first other specialist follow-up (days), HR^b^ | | 1.05 (0.98,1.13) | 1.02 (0.95,1.10) | 1.10 (1.05,1.15)* | 1.03 (0.97,1.08) | 1.17 (1.07,1.26) | 1.06 (0.98,1.16) |
| Other specialist follow-up within 30 days, OR^b^ | | 1.39 (1.03,1.89)* | 1.54 (1.08,2.19)* | 0.86 (0.66,1.12) | 0.80 (0.60,1.07) | 0.78 (0.39,1.59) | 0.69^d^ |
| Length from end of ED presentation to admission, beta coef^c^ | | -0.41 (-1.19,0.36) | -0.30 (-1.06,0.47) | 0.16 (-1.02,1.34) | 0.14 (-1.11,1.39) | -1.17 (-6.64,4.30) | -4.33 (-9.01,0.36) |
| Length of hospital admission (days), beta coef^c^ | | 0.12 (-0.40,0.65) | 0.07 (-0.47,0.61) | 0.12 (-0.19,-0.42) | 0.02 (-0.31,0.35) | 0.11 (-0.93,1.15) | 1.54 (0.25,2.82) |

*p<0.05; a: Patient PIA; b: Discharged patients only; c: Admitted patients only; d: no CIs due to not enough data.

Table S3. Regression estimates for linear models (beta coefs), odds ratios for logistic models (ORs), and hazard ratios for Cox proportional hazard models (HRs) with associated 95% confidence intervals (CIs) for Emergency Department (ED) crowding metric ED length of stay (LOS) for each outcome and each Canadian Triage and Acuity Score (CTAS) group. Adjusted estimates are adjusted for age, sex, income quintile, community size, patient’s zone of residence, weekday, month of year, time of shift, fiscal year, ED category, and Comorbidities (i.e., mild/moderate/severe liver disease, diabetes mellitus, cancer or metastatic solid tumor, myocardial infarction, congestive heart failure, renal disease, peripheral vascular disease, stroke).

| **Outcome** | | **High Acuity (CTAS 1/2)** | | **Moderate Acuity (CTAS 3)** | | **Low Acuity (CTAS 4/5)** | |
| --- | --- | --- | --- | --- | --- | --- | --- |
|  | | Unadjusted (95% CI) | Adjusted (95% CI) | Unadjusted (95% CI) | Adjusted (95% CI) | Unadjusted (95% CI) | Adjusted (95% CI) |
| PIA (hrs), beta coef | | 0.07 (0.06,0.09)* | 0.06 (0.05,0.07)* | 0.25 (0.24,0.26)* | 0.21 (0.20,0.22)* | 0.34 (0.32,0.36)* | 0.30 (0.28,0.32)* |
| ED LOS (hrs), beta coef | | 1.10 (1.00,1.21)* | 1.08 (0.98,1.19)* | 0.92 (0.77,0.86)* | 0.78 (0.73,0.82)* | 0.68 (0.63,0.72)* | 0.59 (0.54,0.64)* |
|  | Discharged^a^ | 0.48 (0.44,0.53)* | 0.43 (0.38,0.48)* | 0.54 (0.52,0.56)* | 0.46 (0.44,0.49)* | 0.58 (0.55,0.61)* | 0.48 (0.45,0.52)* |
|  | Admitted^b^ | 0.78 (0.54,1.02)* | 0.79 (0.55,1.04)* | 0.88 (0.55,1.20)* | 0.90 (0.53,1.26)* | 1.14 (0.11,2.18)* | -0.04 (-0.86,0.79) |
| Disposition, OR | | | | | | | |
|  | Admitted | 1.32 (1.25,1.38)* | 1.28 (1.23,1.34)* | 1.47 (1.34,1.61)* | 1.47 (1.32,1.64)* | 1.43 (1.05,1.96)* | 1.91 (0.83,4.39) |
|  | Left without completion of care | 0.99 (0.82,1.19) | 0.94 (0.75,1.17) | 1.09 (1.00,1.16) | 1.06 (0.95,1.18) | 0.74 (0.55,0.99)* | 0.62^d^ |
| Time to ED return (days), HR^a^ | | 0.98 (0.95,1.01) | 1.00 (0.96,1.03) | 0.96 (0.94,0.98)* | 0.97 (0.95,1.00)* | 0.98 (0.95,1.02) | 1.03 (0.99,1.07) |
| ED return within 30 days, OR^a^ | | 0.92 (0.81,1.03) | 0.92 (0.82,1.04) | 0.92 (0.84,0.99)* | 0.91 (0.83,0.99)* | 0.90 (0.74,1.10) | 0.91 (0.72,1.16) |
| Time to first physician follow-up (days), HR^a^ | | 1.02 (1.00,1.04)* | 1.00 (0.98,1.02) | 1.02 (1.01,1.03)* | 1.00 (0.99,1.02) | 1.06 (1.04,1.08)* | 1.03 (1.01,1.05)* |
| Physician follow-up, OR^a^ | | | | | | | |
|  | Within 7 days | 1.05 (1.01,1.09)* | 1.02 (0.97,1.06) | 1.05 (1.03,1.07)* | 1.03 (1.00,1.05) | 1.11 (1.07,1.15)* | 1.05 (1.01,1.10)* |
|  | Within 14 days | 1.05 (1.02,1.09)* | 1.01 (0.97,1.05) | 1.04 (1.02,1.07)* | 1.02 (0.99,1.04) | 1.11 (1.07,1.15)* | 1.04 (1.00,1.09)* |
|  | Within 30 days | 1.04 (1.00,1.09)* | 1.00 (0.95,1.04) | 1.05 (1.03,1.08)* | 1.02 (0.99,1.05) | 1.13 (1.09,1.17)* | 1.05 (1.00,1.09)* |
| Time to first respiratory medicine specialist follow-up (days), HR^a^ | | 1.05 (1.02,1.08)* | 1.00 (0.97,1.04) | 1.06 (1.04,1.08)* | 1.03 (1.01,1.06)* | 1.10 (1.07,1.13)* | 1.03 (0.98,1.09) |
| Respiratory medicine specialist follow-up within 30 days, OR^a^ | | 1.11 (0.97,1.27) | 1.12 (1.01,1.40)* | 0.95 (0.84,1.08) | 0.89 (0.77,1.03) | 1.14 (0.94,1.38) | 1.03 (0.74,1.42) |
| Time to first other specialist follow-up (days), HR^a^ | | 1.06 (1.01,1.09)* | 1.02 (0.98,1.06) | 1.05 (1.03,1.07)* | 1.02 (0.99,1.04) | 1.12 (1.08,1.15)* | 1.05 (1.00,1.10)* |
| Other specialist follow-up within 30 days, OR^a^ | | 0.96 (0.81,1.14) | 0.97 (0.79,1.18) | 1.04 (0.93,1.17) | 0.97 (0.84,1.13) | 1.05 (0.75,1.46) | 0.85^d^ |
| Length from end of ED presentation to admission, beta coef^b^ | | 0.59 (0.37,0.81)* | 0.67 (0.44,0.90)* | 0.68 (0.38,0.99)* | 0.76 (0.42,1.10)* | 1.19 (0.22,2.16)* | -0.16 (-0.90,0.58)* |
| Length of hospital admission (days), beta coef^c^ | | -0.03 (-0.17,0.12) | -0.03 (-0.19,0.13) | 0.02 (-0.06,0.10) | -0.02 (-0.11,0.07) | 0.00 (-0.20,0.19) | -0.08 (-0.29,0.16) |

*p<0.05; a: Discharged patients only; b: Admitted patients only; c: Admitted patients only; d: no CIs due to not enough data.
